# Estimating the spatial correlation and convergence of China's healthcare resources allocation: evidence from the Yangtze River Delta Region

**DOI:** 10.1186/s13690-022-00958-4

**Published:** 2022-09-14

**Authors:** Yuqing Shen, Zesheng Sun

**Affiliations:** School of Finance and Business, Shanghai Normal University - Shanghai, Shanghai, China

**Keywords:** Yangtze River Delta, Healthcare resources, City clusters, Spatial effects, Convergence, Healthcare reform, China

## Abstract

**Background:**

China’s imbalanced allocation of healthcare resources mainly arises from urban–rural and intercity differences, the solution of which has been the goal of reforms during the past decades. Estimating the spatial correlation and convergence could help to understand the impact of China’s fast-evolving medical market and the latest healthcare reforms.

**Methods:**

The entropy weight method was used to construct a healthcare resource supply index (HRS) by using data of 41cities in a cluster in the Yangtze River Delta (YRD) from 2007 to 2019. The Dagum Gini coefficient, kernel density estimation, Moran's *I*, and LISA cluster map were used to characterize the spatiotemporal evolution and agglomeration of healthcare resources, and then a spatial panel model was used to perform *β* convergence estimation by incorporating the spatial effect, city heterogeneity, and healthcare reforms.

**Results:**

Healthcare resources supply in the YRD region increases significantly and converges rapidly. There is a significant spatial correlation and agglomeration between provinces and cities, and a significant spatial spillover effect is also found in *β* convergence. No evidence is found that the latest healthcare reforms have an impact on the balanced allocation and convergence of healthcare resources.

**Conclusion:**

China’s long-term investment in past decades has yielded a more balanced allocation and intercity convergence of healthcare resources. However, the latest healthcare reforms do not contribute to the balanced allocation of healthcare resources from the supply-side, and demand-side analysis is needed in the future studies.

## Highlights

⦁ This paper provides a more comprehensive measurement of the supply of healthcare resources by using the entropy weight method and the three dimensions of financial, human, and material resources.

⦁ This study suggests that more attention should be given to the spatial interaction among different regions when discussing healthcare resources allocation.

⦁ A spatiotemporal distribution and convergence regression methodology is used to perform healthcare equity research so as to portray the characteristic facts, evolution, and influencing factors of inter-regional healthcare resources allocation.

⦁ Healthcare reforms could be added to convergence spatial panel model estimations, which provides evidence on the possible policy effects of related reforms.

## Background

The unequal allocation of medical resources is an important global challenge [1]. Under the exogenous impact of the Corona Virus Disease 2019 (COVID-19) pandemic, this means a serious inequality in the right to survival and health. Investment in healthcare is becoming a high public health priority globally. Most growth in both real GDP and healthcare expenditures worldwide is occurring in emerging Eurasian countries, mainly represented by the Emerging Markets Seven (EM7), Brazil, Russia, India, China, and South Africa (BRICS), and other emerging markets [2]. Healthcare reforms in these emerging markets are affecting the pattern of global healthcare expenditure, which is dominantly attributable to the reforms carried out in China [3]. However, it remains to be explored whether the increase in healthcare expenditure occurring in emerging markets promotes a domestic balance of resource allocation among different regions in those countries, and better understanding is still needed regarding the spatial correlation that such expenditure growth has on resource allocation among different internal regions.

After the severe acute respiratory syndrome (SARS) crisis broke out in 2003, the Chinese government has begun to allocate more budget to grassroots (county/township) hospitals in order to rebuild the grassroots public health system, which was severely weakened due to the long-term market reform [4]. In March 2009, China launched a new round of healthcare reforms, clearly proposing that the increased healthcare expenditures should be focused on demand side, public health, and the grassroots. But these reforms tended to pay more attention to medical infrastructure hardware, and less attention to medical staff and their human capital. Therefore, patients are still more willing to choose to go to (urban) high-level hospitals for treatment, creating a structural congestion problem in which high-level hospital congestion and idle low-level hospital resources coexist [5].

After 2010, some of China's medical reform pilot provinces began to implement the descending healthcare resources reform represented by the combination of descending doctors, the spillover of human capital from high-level hospitals, and brand placement in low-level hospitals, so as to enhance the latter’s diagnosis and treatment capabilities, attract human capital inflows, and reshape patient expectations. In 2015 and 2017, this reform received the attention of the Chinese government and was promoted nationwide [6, 7]. Meanwhile, starting in 2015, some pilot provinces and cities, including Shanghai and Zhejiang, has begun to implement comprehensive medical reform focusing on medical prices, medical insurance, and pharmaceutical procurement to reduce medical costs and establish a more effective hierarchical diagnosis and treatment system. However, whether the past reforms have resulted in a more balanced allocation of healthcare resources and generated the convergence effect is still a question to be answered.

Previous literatures have used neoclassical convergence theory to estimate the convergence of healthcare expenditure dynamics. The main research perspective is multi-country convergence estimation and comparison. Studies on the European Union (EU), Organization for Economic Co-operation and Development (OECD), and other economies have found that healthcare expenditures of EU countries have statistically significant σ convergence and β convergence characteristics [8], and the per capita healthcare expenditure of OECD countries has club convergence [9]. However, no single equilibrium in healthcare expenditure exists for sub-Saharan African countries, although three convergence clubs have been identified [10]. Other studies delved into different regions within individual countries, but also mainly used healthcare expenditure as an indicator. Studies have confirmed the existence of convergence of healthcare expenditure in US states [11], and further found that Indian states can be divided into two convergence clubs for healthcare expenditures [12]. In addition, healthcare expenditures of all counties in China tend to converge [13]. However, healthcare resource allocation is not limited to healthcare expenditure indicators, but should also include other indicators of healthcare resources supply, such as numbers of healthcare workers and beds. Although a few studies have discussed the convergence of single indicators such as numbers of beds and doctors [14], our literature review found no studies that utilized the comprehensive healthcare resource supply index, including dimensions of human, material, and financial resources, and performed corresponding convergence estimations.

Existing studies have recognized that the imbalanced healthcare allocation in China is the most serious and urgent public service challenge [15, 16]. Under the administrative hierarchy, high-level hospitals receive more government financial resources, and thus can attract higher quality human capitals [17]. The resulting lack of service capacity, and patients’ disapproval and distrust further weaken low-level hospitals’ ability to attract patients [18]. The 2009 healthcare reform was believed to accelerate the convergence rate of provincial healthcare resource supply in China, but it is still slower than that of per capita Gross Domestic Product (GDP) [19]. Furthermore, some studies have found that the imbalance continues to be serious. One explanation focuses on the substantial imbalance of material and financial resources among regions [20]. Another explanation is related to the 2009 healthcare reform, suggesting that the expanded coverage of medical insurance relaxed patients’ financial constraints, which prompted them to choose high-level hospitals [21]. However, existing studies have not discussed the imbalance and convergence in the context of the latest healthcare reforms and the reform effects.

Some studies estimated the spatial distribution of healthcare resources using different measures, including Gini coefficient [22, 23], to analyze the dynamics of healthcare resources supply and the reform effects in both time and space dimensions with mainly provincial-level data. The problem is that regional differences in healthcare resources supply often occur within one province, especially among provincial capitals, core cities, and peripheral cities dominated by rural areas. Therefore, provincial-level estimation cannot accurately reflect the urban–rural differences in healthcare resource allocation. Meanwhile, less attention is paid to the spatial correlation, heterogeneity of cities of different sizes/levels, and the impact of the latest reforms. Although a few studies have used hospital- and patient-level data to evaluate the effects of reform policies, such as the descending resources reform and medical treatment combination, and provided evidence of their effects in incentivizing patients to choose low-level hospitals and improving the efficiency of resource allocation [24, 25], it is not clear if these reforms have generated macro-level convergence effects among cities and regions.

Since the spatial correlation of healthcare resource allocation mainly occurs between neighboring cities, city clusters could be a suitable research object to discuss the spatial correlation and convergence of healthcare resources supply. This paper focuses on the city cluster in the Yangtze River Delta (YRD), which includes Shanghai, Jiangsu, Zhejiang, and Anhui. In the *Outline of the Integrated Regional Development of the Yangtze River Delta* proposed by the Chinese government [26], a more balanced distribution of healthcare resources was listed as a regional integration goal. Moreover, the data availability of this region is better than that of other regions in China. We collected data of 41 YRD cities at and above the prefectural level from 2007 to 2019 to perform the empirical study. The sample includes Shanghai, which has a population of about 25 million, and Hangzhou, Suzhou, Nanjing, Hefei and Ningbo, which have a population of nearly 10 million each, as well as prefecture-level cities with vast rural regions. The scope and specific cities of the Yangtze River Delta are shown in Fig. 4 in Appendix.

Compared with the existing literature, this paper makes the following novel contributions: (1) This paper incorporates the spatial correlation in the YRD city cluster into the analysis of healthcare resource allocation, and for the first time provides evidence on China’s healthcare resource allocation with a representative cluster of 41 cities at and above the prefectural level. (2) This paper combines the measurement of spatiotemporal distribution with the convergence estimation, which enables us to understand the characteristic facts of healthcare resources allocation in the YRD region, and also incorporates city heterogeneity into the analysis. (3) It is the first attempt to estimate the convergence effects of the latest reforms, in which the descending healthcare resources reform and comprehensive medical reform are included in the empirical estimation.

The structure of this paper is as follows: Reviews the literature,  presents the methods and data used in this paper,  report the characteristic facts and the convergence estimation results, and the last section concludes.

## Literature review

The balanced allocation of healthcare resources aims to match the supply side with the demand side, thereby helping to achieve the equalization of healthcare services across regions [5]. It will also be of vital importance in reducing high-level hospital congestion and the costs of patient diagnosis and treatment. To measure the healthcare resources allocation, two approaches could be used. The first approach is to observe the cross-sectional spatial distribution with a number of measurement indicators. If the time dimension is further introduced, the evolutionary trend could also be discussed [27]. If different cross-sectional regions are included with geographic information system (GIS), we could intuitively understand the trend of spatiotemporal evolution [28]. However, because of the existence of cross-sectional heterogeneity, measurement indicators cannot control for the influence of heterogenous factors, so we cannot capture the spatiotemporal evolution characteristics in a comparable way for different regions [29]. Therefore, another approach uses regression techniques to control for the impact of heterogeneous subjects. The convergence estimation technique developed for economic growth research could be introduced to estimate the convergence of healthcare resource allocation [30]. Three branches of literature are relevant to this study.

The first branch of literature generally uses one or more measurement indicators including the agglomeration index, coefficient of variation, Gini coefficient, and Theil index for analysis [31, 32]. Among these, the agglomeration index is used to measure the ratio of population or geographic area in a specific region to the amount of existing health resources. Although this can reflect the balanced degree of health resource allocation among different groups in the same region [33], it cannot reveal the balanced degree of resource allocation within the group. In contrast, the coefficient of variation, i.e., the ratio of the standard deviation of the healthcare resource supply level within a specific area to its arithmetic mean, can better measure the balanced degree of resource allocation within the group [34].

Due to the lack of objective evaluation benchmarks for the aforementioned two indicators, academics also use the Gini coefficient and Theil index with a definite assignment range (∈ [0,1]) and objective evaluation benchmark for estimation. The early Gini coefficient was used to reflect the income inequality of residents [35], but it could not reflect the overall internal balance. Therefore, Dagum [36] proposed the decomposable Gini coefficient, which can decompose overall regional differences according to subgroups, and effectively solve the overlapping problems among observed samples. The Theil index is obtained by the entropy (or grouping entropy) method [37, 38], but the impact of geographical accessibility is not considered [39]. Due to the multi-input and multi-output characteristics of healthcare resources, one method is to use data envelopment analysis (DEA) to measure the relative efficiency of a group of decision-making units with multi-inputs and multi-outputs [40, 41]. An alternative method is to use the entropy weight method to construct the supply/demand index of health resources based on multi-input and multi-output data [42].

Based on the above measurement indicators, the existing empirical literature on healthcare resource allocation in China has mainly focused on the whole country or a single administrative region as the research object. Estimations based on agglomeration index have shown that the degree of healthcare resource agglomeration in economically developed provinces/cities is relatively high, whereas the geographical accessibility of healthcare resources in economically underdeveloped provinces/cities is poor [43]. There are obvious differences in heath resource allocation in different provinces, such as Sichuan [44], Zhejiang [45], and Henan [46], which have different characteristics of agglomeration based on population size and geographic area. Yi et al. (2020) measured the coefficient of variation and found no *σ* convergence in eastern, central, and western China; that is, healthcare efficiency differences within China may gradually expand [47]. Another estimate based on Dagum Gini coefficient, however, shows that the overall, intra-regional and inter-regional differences in the supply of basic medical and health services decreased during 2007–2018 [22]. In addition, the Theil index has been used to demonstrate that resource allocation variations in different provinces are attributable to different sources. For example, differences in healthcare resource allocation in Anhui province are mainly caused by intra-regional differences [48], whereas those in Hainan Province are caused by inter-regional differences [49].

The second branch of research explores the spatial correlation of healthcare resource allocation by using GIS or other spatial analysis tools. The advantage is that it allows to visually represent the differences in the spatial distribution of healthcare resources. There are three commonly used spatial analysis methods. The exploratory spatial data analysis method (ESDA) mainly uses the Moran’s *I* to analyze the spatial correlation of the economic and social attributes of the spatial unit [50], and can also use a LISA cluster map to present the agglomeration characteristics of a specific area. Kernel density estimation (KDE) approximates the probability density of the sample to visualize the spatial points of the characterizing area [51]. The KDE method can present the spatial distribution of the whole area, which is complementary to the results of ESDA analysis. The standard deviation ellipse (SDE) method constructs a standard deviation ellipse to reflect the dominant distribution direction of spatial elements and the dispersion of each direction, which, like the LISA cluster map, can make up for the fact that the KDE map cannot display the distribution characteristics of specific regions [52].

Spatial statistical analysis can be carried out from different levels such as the whole country, city clusters, and individual cities. From the national dimension, KDE analysis revealed that China’s inter-provincial healthcare resources supply tends to rise; however, polarization can be found in both the national and eastern regions, while there is no gradient effect in the central and western regions [22]. Very few studies focused on the dimension of prefecture-level cities, and the results of Moran's *I* and LISA cluster map showed that the spatial correlation pattern of healthcare resources supply changed greatly; and the central, western, and northeast regions showed high-high agglomeration characteristics [53]. However, other studies reported that the spatial distribution of healthcare resources among cities tends to be balanced, and there are low-low agglomeration regions with a concentrated distribution in the transition zone from the eastern coast to the central region [28]. The above empirical research using prefecture-level city data adopted a similar method, but produced inconsistent results because of differences in the research period and processing methods used for the indicators. Lastly, most spatial statistical studies take specific cities as research objects and use the SDE method to explore the spatial distribution of healthcare resources within cities [23, 54, 55].

The third branch of research includes very few studies that use a panel econometric model to explore the agglomeration or convergence trends of healthcare resources and the influencing factors of spatial differences. According to our survey, most of previous studies used provincial-level panel data to perform the convergence estimation. For instance, Zhou (2018) found conditional *β* convergence, but not absolute *β* convergence between provinces by using single indicators [29]. And the increases of medical expenditure and regional financial autonomy would push regional healthcare resources onto the path of convergence. Pan et al. (2017) confirmed the long-term trend of absolute and conditional *β* convergence in government health spending in China [56]. Considering the multi-input and multi-output characteristics of healthcare resources, some studies used DEA to measure the total factor productivity (TFP) of different provinces and estimated its convergence [57]. It is found that the overall TFP level of provincial health resources in China is low, but the growth of TFP has absolute and conditional *β* convergence and presents regional differences. Although the spatial spillover effect is observed in the *β* convergence of healthcare resource allocation [30], according to our search, no studies have included spatial effect into city-level analysis in the context of the latest healthcare reforms in China.

To sum up, the spatial differences of healthcare resource allocation in China have attracted extensive attention. As is known, the imbalance in healthcare resource allocation in China mainly occurs between urban and rural areas and between core and peripheral areas, and the newly implemented healthcare reforms may produce marginal impacts. Thus, latest research should focus on city clusters within provinces and neighboring provinces, and incorporate city heterogeneity and the possible impact of healthcare reforms. Accordingly, one representative region could be used to provide new evidence for the allocation of healthcare resources and evaluation of the effects of China’s reforms. This paper takes the YRD as the research object and investigates the spatial correlation among cities to intuitively depict the spatial dynamic distribution of healthcare resources with a multi-measure index. The spatial panel model was then used to analyze the convergence of healthcare resources in the YRD region.

## Methods and data

### Methods

- (1) Measurement of spatial distribution of healthcare resources

We first construct a healthcare resources supply index (HRS) using the entropy weight method, analyze regional differences, and then examine the sources of differences by using the Gini coefficient decomposition method given by Dagum [36]. Let *G* represent Gini coefficient, *j* and *h* represent the number of regions, *i* and *r* represent the number of cities in the region, *k* represent the total number of regions, *n* represent the total number of cities, and *n*_*j*_(*n*_*h*_*)* represent the number of cities in region *j*(*h)*. Further, *y*_*ij*_(*y*_*rh)*_ represents the HRS of city *i*(*r)* in region *j*(*h)*, and $\overline{y}$ represents the arithmetic mean of the HRS. The following equation can be obtained:

1 $$G{ = }\frac{{1}}{{2n^{2} \overline{y}}}(\sum\limits_{i=1}^{n} {\sum\limits_{r=1}^{n} {|y_{i} - y_{r} |} } ) = \sum\limits_{j=1}^{k} {\sum\limits_{h=1}^{k} {\sum\limits_{i=1}^{{n_{j} }} {\sum\limits_{r=1}^{{n_{h} }} {|y_{ij} - y_{rh} |/2n^{2} \overline{y}} } } }$$

After the overall Gini coefficient is calculated, *k* regions are sorted according to the average HRS in the YRD, namely *y*_*1*_ ≤ … ≤ *y*_*j*_ ≤ …*y*_*k*_, and then the Gini coefficient *G* is decomposed into three parts: (1) intra-regional difference contribution (*G*_*w*_), (2) inter-regional difference contribution (*G*_*nb*_), and (3) transvariation density contribution (*G*_*t*_), which meet the requirements of *G* = *G*_*w*_ + *G*_*nb*_ + *G*_*t*_.

Next, we use the KDE method to explore the spatial distribution dynamics of the HRS in the YRD. Specifically, we use Moran’s *I* to examine the spatial correlation of the YRD's HRS. If Moran’s *I* is significant, the LISA cluster map will be used to determine whether the local correlation types and clusters in different regions are statistically significant. When Moran's *I* value is [− 1, 0), 0, and (0, 1], it means negative correlation, irrelevance, and positive correlation, respectively. According to the LISA cluster map, four types of spatial agglomeration relationships can be identified: (1) high-high agglomeration area (HH type), (2) high-low agglomeration area (HL type), (3) low-low agglomeration area (LL type), and (4) low–high agglomeration area (LH type).

- (2) Convergence estimation of healthcare resource allocation

Both *σ* convergence estimation and *β* convergence estimation are commonly used in convergence analysis [58]. The former estimate does not rely on an econometric model and uses the coefficient of variation to measure, which can confirm the previous Gini coefficient results. *β* convergence can be divided into absolute *β* convergence and conditional *β* convergence. The former is used to assess whether the supply of healthcare resources in various regions will converge to the same steady-state equilibrium point without considering the city heterogeneity factors. More importantly, we include spatial factors, heterogeneity, and healthcare policy as control variables in conditional *β* convergence for a more comprehensive estimation.

The model for absolute *β* convergence is set as follows:

2 $$\ln (\frac{{y_{i,t+1} }}{{y_{i,t} }}) = \alpha + \beta \ln (y_{i,t} ) + \mu_{i} + \eta_{t} + \varepsilon_{it}$$

where *i* represents the city; *t* represents the time; *y*_*i,t*+1_ and *y*_*i,t*_ represent the HRS of city *i* in *t* + 1 period and *t* period, respectively; ln(*y*_*i,t*+1_*/y*_*i,t*_) represents the annual growth rate of HRS in city *i* during the period from *t* to *t* + *1*; and *β* is the convergence parameter to be estimated, where *β* < 0 means an absolute *β* convergence trend, and otherwise it indicates that there is a divergence trend. *α* is a constant term; *μ*_*i*_ and *η*_*t*_ represent regional and time effects, respectively; and *ε*_*it*_ represents the random interference term. The formula of convergence rate is $v = - \frac{1}{TS}\ln (1 + \beta )$, where *TS* represents the time span.

If heterogeneous factors such as economic development status, city size, and government fiscal capacity are included in the model, the model for conditional *β* convergence is set as follows:

3 $$\ln (\frac{{y_{i,t+1} }}{{y_{i,t} }}) = \alpha + \beta \ln (y_{i,t} ) + \gamma \ln (X_{i,t} ) + \mu_{i} + \eta_{t} + \varepsilon_{it}$$

where *X*_*i,t*_ is the control variable; *γ* is the parameter to be estimated for the control variable; and the meaning of the other variables is the same as the formula (2).

Considering the possible impact of policies and external shocks on the conditional *β* convergence, we have:

4 $$\ln (\frac{{y_{i,t+1} }}{{y_{i,t} }}) = \alpha + \beta \ln (y_{i,t} ) + \gamma \ln (X_{i,t} ) + \delta P_{i,t} + \tau [\ln (y_{i,t} ) \times P_{i,t} ] + \mu_{i} + \eta_{t} + \varepsilon_{it}$$

where *P*_*i,t*_ is a policy dummy variable, where if region *i* starts to implement the policy in period *t*, then *P*_*i,t*_ is assigned a value of 1 during period *t* and after, and *P*_*i,t*_ is assigned a value of 0 before period *t*; and *δ* represents the impact of the policy on the growth rate of HRS; *τ* represents the impact of policy on the convergence of HRS.

- (3) Spatial panel model

Taking into account the spatial correlation, we construct a spatial econometric model of *β* convergence. In order to determine the exact form of the spatial effects entering the panel model, we first estimate the following three spatial models in the absolute *β* convergence estimation:

5 $$\ln (\frac{{y_{i,t+1} }}{{y_{i,t} }}) = \alpha + \beta \ln (y_{i,t} ) + \rho \sum\limits_{j=1}^{N} {W_{ij} \ln (\frac{{y_{i,t+1} }}{{y_{i,t} }})} + \mu_{i} + \eta_{t} + \varepsilon_{it}$$

6 $$\ln (\frac{{y_{i,t+1} }}{{y_{i,t} }}) = \alpha + \beta \ln (y_{i,t} ) + \mu_{i} + \eta_{t} + \varepsilon_{i,t} ,\varepsilon_{i,t} = \lambda \sum\limits_{j=1}^{N} {W_{ij} \varepsilon_{j,t} } + \sigma_{i,t}$$

7 $$\ln (\frac{{y_{i,t+1} }}{{y_{i,t} }}) = \alpha + \beta \ln (y_{i,t} ) + \rho \sum\limits_{j=1}^{N} {W_{ij} \ln (\frac{{y_{i,t+1} }}{{y_{i,t} }})} + \theta \sum\limits_{j=1}^{N} {W_{ij} \ln (y_{i,t} )} + \mu_{i} + \eta_{t} + \varepsilon_{it}$$

Equation (5) is the spatial autoregressive *β* convergence model (SAR), and Eqs. (6) and (7) are the spatial error *β* convergence model (SEM) and the spatial Dubin *β* convergence model (SDM), respectively. In these equations, *ρ* represents the spatial effect coefficient of the explained variable, reflecting the influence of the explained variable in the neighboring cities; *λ* represents the spatial effect coefficient of the error term, reflecting the random shock; *θ* represents the spatial effect coefficient of the explanatory variable, reflecting the influence of the explanatory variable of neighboring cities; and *W*_*ij*_ represents the spatial weight matrix. In this paper, we use the inverse distance weight matrix.

After estimating the three above spatial panel models, following Alhorst [59] and Han [60], we test and select appropriate spatial econometric models. First, the Lagrange multiplier (LM) test is performed based on the traditional panel model. If the LM test is significant, the SDM model will be used. Then, the likelihood ratio (LR) test and Hausman test are used to determine individual/time effects and fixed/random effects. Subsequently, the Wald and LR tests are used to determine whether the SDM model can be degraded to the SEM or SAR model. If the degraded result is inconsistent with the LM test result, the SDM model will be selected. In the conditional *β* convergence estimation, we also use the same strategy to determine the specific form of the spatial econometric model.

### Data and variables

According to the State Council (2019) [26], the Yangtze River Delta (YRD) region includes Shanghai (SH), Jiangsu (JS), Zhejiang (ZJ), and Anhui (AH), consisting of 41 cities at and above the prefectural level. The main variables and data used in this study are reported below.

- (1) Healthcare resource supply index (HRS)

Because healthcare resources include human, material, and financial resources [11], two indicators are selected for each of the above three aspects according to the availability of data, and the entropy weight method is used to construct the HRS index [22, 61]. The data source and weight are reported in Table 1. The HRS results of the 41 YRD cities from 2007 to 2019 are reported in Table 11 in Appendix. It can be seen that the HRS of the YRD and sub-provincial regions has shown a continuous upward trend with certain differences among the provinces.

- (2) Control variables and policy variables

**Table 1:** Evaluation index system of HRS

Dimension	Index	Weight
Financial resources	Healthcare expenditure per capita (yuan)	0.272
	Proportion of healthcare expenditure in regional fiscal expenditure (%)	0.140
Material resources	Number of beds per square kilometer	0.233
	Number of beds per 1, 000 persons	0.109
Human resources	Number of practicing (assistant) physicians per 1,000 persons	0.108
	Number of registered nurses per 1,000 persons	0.139

Source: *China Health Statistics Yearbook* from 2006 to 2020, *Zhejiang Health and Family Planning Yearbook* from 2006 to 2020, *Jiangsu Health Family Planning Yearbook* from 2006 to 2020, *Zhejiang Financial Yearbook* from 2006 to 2020, and relevant provincial and municipal health committees over the years

The weight is calculated by the entropy weight method [42]

In order to estimate conditional *β* convergence, it is necessary to control the heterogeneity factors across cities. According to the availability of data, we include the following control variables in the model: city size (SIZE), economic development level (GDP), urbanization level (UR), government financial capacity (GFC), and government financial self-sufficiency rate (GFS) [62–64]. The definitions and data sources of these variables are reported in Table 2. Logarithm processing is used to eliminate the dimensional difference.

**Table 2:** Definition and descriptive statistics of control variables

Variable	Variable definition	Maximum value	Minimum value	Mean	standard deviation
SIZE	Regional resident population (10,000 people)	2428.1	72.0	528.3	369.0
GDP	GDP per capita (yuan)	180,000.0	5515.0	57,950.5	35,919.3
UR	Proportion of urban population (%)	89.6	29.0	58.1	12.8
GFC	Local budgetary expenditure/GDP (%)	35.7	5.7	15.4	6.2
GFS	Local budgetary revenue/expenditure (%)	116.7	19.4	64.5	23.7

Source: Urban Statistical Yearbook of China from 2006 to 2020, Statistical Communique of the People's Republic of China on the National Economic and Social Development from 2006 to 2020, and China Fiscal Yearbook from 2006 to 2020

Because the existence of medical universities/colleges may affect the HRS of that specific city, we differentiate the city subsamples according to the existence of medical universities/schools and medical graduate programs in the starting year of the sample. The medical education data of different cities are reported in Table 12 in Appendix.

We also incorporated the main healthcare reforms into the empirical model. The policy variables include the descending healthcare resources reform (DHR) and comprehensive medical reform (CMR). The descending healthcare resources reform is to encourage the balanced distribution by building medical treatment combinations and increasing government investment [65]. Different cities usually experienced different timing of reforms. We take the time of first document issued by the local health departments (authorities) or the time of the first medical treatment combination being organized as the reform time point (see Table 12 in Appendix). We then define one policy dummy DHR, setting the after-reform period as 1 and the pre-reform period as 0.

The comprehensive medical reform tries to reduce diagnosis and treatment costs and realize the goal of hierarchical diagnosis and treatment. In February 2015, Jiangsu and Anhui were identified as pilot provinces for comprehensive medical reform, followed by Zhejiang and Shanghai in May 2016. The date of publication by local health authorities or the official launch date of the reform is used as the time point of reform, so we have the dummy CMR. It should be noted that, if the above reform is launched before July 1 of the current year, the value of 1 is assigned for the current year, and if the reform is launched after July 1 of the current year, the value of 1 will be assigned from the next year.

## Spatial distribution dynamics of healthcare resources

### Dagum Gini coefficient and σ convergence results

Because we take Shanghai as one city sample on the cross-section, the Dagum Gini coefficient calculation gives the results of the three YRD provinces except Shanghai, but Shanghai is included in the inter-regional difference analysis and difference decomposition. For comparison with the Gini coefficient measurements, we also report the *σ* convergence measurements obtained with the coefficient of variation.

**Fig. 1:**
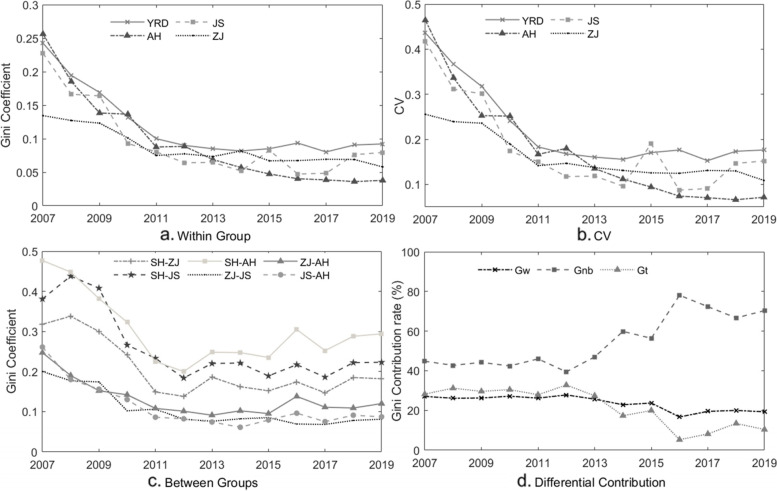
Estimation of the Dagum Gini coefficient and coefficient of variation in the Yangtze River Delta, China, 2007–2019. Source: Calculated by the authors using MATLAB software

As shown in Fig. 1(a), the average Gini coefficient of Zhejiang province is the smallest, with a value below 0.15 in 2007, indicating highly balanced allocation of healthcare resources. In the early stage, Anhui province had the largest average Gini coefficient (> 0.25). Although the Gini coefficients of the three provinces all showed a downward trend in the sample period, Anhui province had the strongest downward trend, with its Gini coefficient reaching its lowest level in 2019 (< 0.05). A similar trend is found when using the coefficient of variation, as reported in Fig. 1(b), indicating a significant *σ* convergence tendency.

Figure 1(c) further reports the results of the Dagum Gini coefficient for the pairwise comparison of four provinces/cities. It can be seen that in the early sample period, the regional difference between Shanghai and Anhui was the largest, while the regional difference between Jiangsu and Zhejiang was the smallest. The differences between Zhejiang and Anhui, Jiangsu and Anhui, and Zhejiang and Jiangsu were all relatively small during the sample period. Moreover, the differences between regions tend to decline before 2012 and then rise with fluctuations. Overall, the differences of healthcare resources allocation in the YRD region declined during the sample period.

To further understand the source of these observed differences, we follow Dagum (2007) to decompose the Gini coefficient into three components [36]. The average annual contribution rates of *G*_*w*_, *G*_*nb*_, and *G*_*t*_ are 23.7%, 54.62%, and 21.68%, respectively. It can be seen from Fig. 1(d) that the contribution rate of inter-regional differences increased rapidly after 2012, and exceeded 70% by 2019. The contribution rate of intra-regional differences decreased slowly, but the contribution rate of transvariation intensity, which measures the interaction between inter-regional differences and intra-regional differences, decreased rapidly and reached nearly 10% in 2019. This suggests that we should pay more attention to the spatiotemporal distribution among provinces and city groups.

## Kernel density curve estimates

**Fig. 2:**
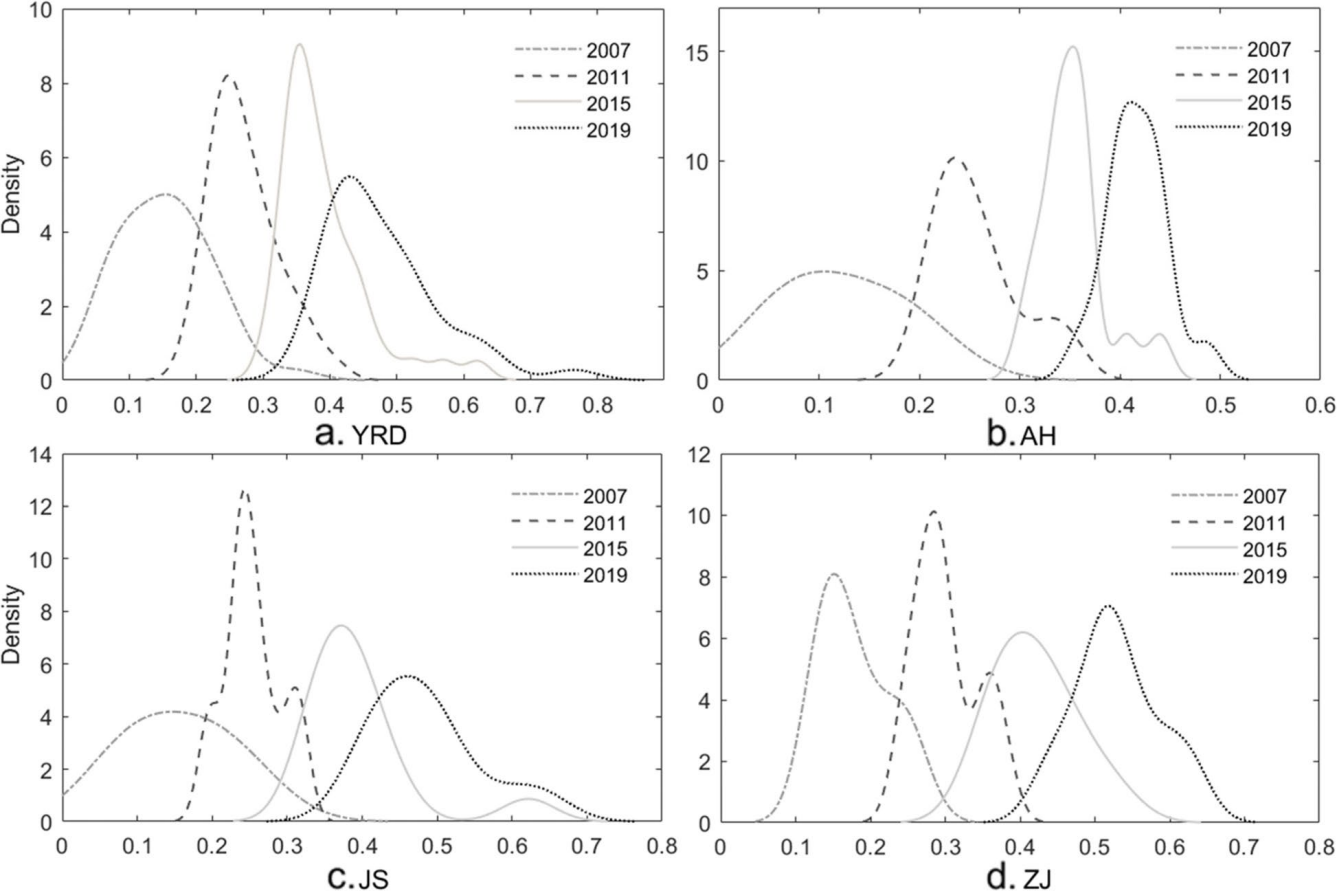
Kernel density distribution in the Yangtze River Delta, China, 2007–2019. Source: Created by the authors using MATLAB software

In order to further capture the spatial dynamics of healthcare resource allocation within the YRD provinces, we used the KDE method to present the kernel density curves of HRS in selected years (Fig. 2). The center of the kernel density curve gradually moved to the right, indicating that HRS in the YRD and related provinces increased significantly. According to the distribution morphology in Fig. 2(a), the height of the kernel density curve of the YRD evolved by increasing first, and decreasing then, narrowing first and widening then. These changes indicate that the dispersion degree of the YRD first decreased and then increased. The same trend is found in Anhui and Jiangsu, while the dispersion degree of Zhejiang has little change. From the perspective of distributional ductility, the right tail of the kernel density curve indicates that cities with a higher supply of healthcare resources would experience faster growth, as highlighted in Fig. 2(a) and (c). In particular, Jiangsu exhibits a left-tailing phenomenon, indicating that its internal slowdown and acceleration may coexist. The bimodal and multimodal kernel density curves, correspond to internal polarity and multipolar differentiation trends, respectively. In the sample period, obvious differentiation could no longer be found in the YRD and the three provinces by 2019. To sum up, all provinces, including the YRD, experienced the evolution of healthcare resource allocation from decentralization to agglomeration and then to decentralization. Among them, Anhui exhibits the most obvious agglomeration feature, which is similar to the results in Dagum Gini Coefficient and σ Convergence Results section.

## Spatial agglomeration dynamics

In order to investigate the spatial correlation of HRS in the YRD, Moran's *I* was first estimated, and then a LISA cluster map was used to visually display the agglomeration dynamics among cities. As shown in Table 3, Moran's *I* values are all greater than 0 at the significance level of 5%. The significantly positive value of Moran's *I* indicates that a significant positive spatial correlation exists during the sample period, which provides evidence for the inclusion of spatial factors in this study.

**Table 3:** Estimation of Moran's *I* in the Yangtze River Delta, China, 2007–2019

Year	Moran's *I*	Z value	*P* value
2007	0.111	6.189	0.000
2008	0.074	4.673	0.000
2009	0.074	4.648	0.000
2010	0.039	2.935	0.003
2011	0.039	2.93	0.003
2012	0.022	2.127	0.033
2013	0.025	2.314	0.021
2014	0.056	3.795	0.000
2015	0.045	3.311	0.001
2016	0.122	6.887	0.000
2017	0.090	5.356	0.000
2018	0.064	4.173	0.000
2019	0.084	5.103	0.000

Calculated by the authors using GeoDa software

**Fig. 3:**
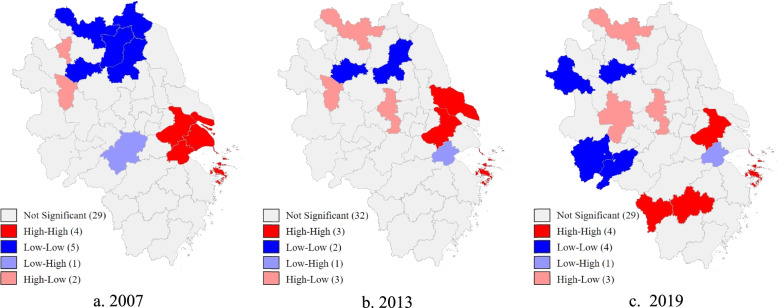
LISA cluster maps of healthcare resource supply in the Yangtze River Delta, China, 2007–2019. Source: Created by the authors using GeoDa software

**Fig. 4:**
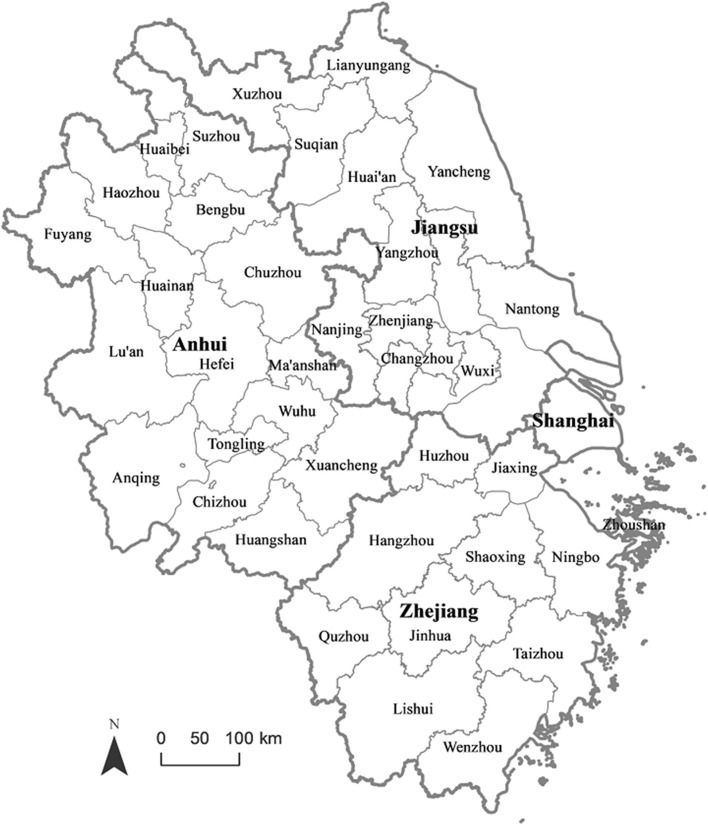
The scope of the Yangtze River Delta in China. Source: The authors

In order to more accurately reveal the spatial correlation and agglomeration characteristics in the YRD, GeoDa software is used to draw LISA cluster maps and visualize the agglomeration and diffusion characteristics of the YRD cities. The LISA cluster results are reported in Table 13 in Appendix. Figure 3 shows that healthcare resources allocation in the YRD exhibit a agglomeration effect. The high-high agglomeration area has shifted from the mid-east region of the YRD to the south. Suzhou has kept its position as the high-high agglomeration core city, but that of Shanghai has gradually declined. By 2019, the three other high-high agglomeration core cities except Suzhou are all located in Zhejiang. Meanwhile, low-low agglomeration regions have gradually shifted from Jiangsu to Anhui. By 2019, the core cities of low-low agglomeration were all located in Anhui, while the core city of the low–high agglomeration area has changed from Xuancheng, Anhui, to Jiaxing, Zhejiang. Meanwhile, the high-low agglomeration regions have shifted from Huaibei and Huainan, and Nanjing and Xuzhou have particularly prominent high-low agglomeration characteristics. In summary, during the sample period, low-low agglomeration regions have gradually shifted from Jiangsu to Anhui, Zhejiang gains more high-high agglomeration regions, and Jiangsu has a stable high-high agglomeration state.

## Panel model estimation results

### Estimation results of the benchmark model

Before the panel model estimation, we first need to determine the exact form in which spatial effects enter the panel model. The results in Table 4 suggest that LM and Robust LM tests reject null hypothesis at the significance level of 5% in both absolute *β* convergence and conditional *β* convergence estimates, indicating the existence of spatial effects. The LR tests showed that both individual and time effects exist, and spatial Hausman test results suggest that we should use the fixed-effect spatial panel model. The LR test and Wald test of absolute *β* convergence showed that SDM could degenerate into SAR or SEM, and pointed to SEM in LM and Robust LM tests, so the two-way fixed effects SEM model was selected for absolute *β* convergence estimation. LR and Wald tests in conditional *β* convergence showed that SDM could not degenerate into SAR or SEM, and lag terms and error terms were significant in LM and Robust LM tests. Therefore, the two-way fixed-effects SDM model was selected for conditional *β* convergence estimation.

**Table 4:** Test results of the spatial β convergence model

Test term	Space absolute β convergence	Space conditional β convergence
LM spatial lag	43.395*** (0.000)	86.524*** (0.000)
Robust LM spatial lag	37.525*** (0.000)	6.285** (0.012)
LM spatial error	230.762*** (0.000)	184.763*** (0.000)
Robust LM spatial error	224.891*** (0.000)	104.524*** (0.000)
LR test ind	18.48*** (0.005)	25.65** (0.029)
LR test time	122.52*** (0.000)	109.72*** (0.000)
Spatial Hausman	24.35*** (0.000)	50.49*** (0.000)
LR test spatial lag	0.34 (0.558)	15.17** (0.019)
Wald test spatial lag	0.36 (0.550)	14.52** (0.013)
LR test spatial error	0.10 (0.749)	17.10*** (0.009)
Wald test spatial error	0.16 (0.689)	17.36*** (0.008)
Selected Model	SEM	SDM

(a) ***, **, * indicate significance at the 1%, 5%, and 10% levels, respectively, and standard errors are in parentheses. (b) Estimated using Stata software

Using Eqs. (2) and (3) for regression, in Table 5, we first report the *β* convergence results without including spatial effects. It can be seen that the regression coefficients *β* of HRS in model (1) and model (3) are both below zero and statistically significant, indicating that the growth rate of HRS is negatively correlated with the initial equalization level; that is, there is absolute *β* convergence and conditional *β* convergence, with a convergence rate of 5.31% and 8.22%, respectively. Next, we incorporate the spatial effect into the estimation, as shown in Table 5. According to the estimation results of model (2) and model (4), the healthcare resource supply in the YRD region still significantly converges, with a rate of 5.27% and 8.61%, respectively. This result provides support for the estimation results of model (1) and model (3). At the same time, the estimation results of *ρ*/*λ*, which is a parameter describing the spatial effects, show that the spatial effects are significantly positive, indicating that the spatial spillover effect will accelerate the healthcare resource supply in the neighboring cities. This conclusion is consistent with the results reported in Sect. 4.

**Table 5:** Empirical results of *β* convergence

Variable	(1) Absolute β convergence	(2) Spatial absolute β convergence	(3) Conditional β convergence	(4) Spatial conditional β convergence
	two-way fixed effects OLS	two-way fixed effects SEM	two-way fixed effects OLS	two-way fixed effects SDM
β	-0.471^***^ (0.027)	-0.469^***^ (0.026)	-0.627^***^ (0.055)	-0.644^***^ (0.033)
ln_SIZE			-0.069 (0.072)	-0.030 (0.061)
ln_GDP			0.213^**^ (0.094)	0.236^***^ (0.066)
ln_UR			0.193 (0.123)	0.175^**^ (0.074)
ln_GFC			0.134^**^ (0.050)	0.151^***^ (0.047)
ln_GFS			-0.011 (0.042)	0.073 (0.051)
W × β				-0.067 (0.270)
W × ln_SIZE				-0.829^*^ (0.487)
W × ln_GDP				0.584 (0.464)
W × ln_UR				0.172 (0.599)
W × ln_GFC				0.097 (0.308)
W × ln_GFS				-1.156^***^ (0.395)
ρ/λ		0.345^**^ (0.156)		0.307^*^ (0.162)
N	492	492	492	492
R^2^	0.527	0.284	0.580	0.010
Convergence rate	5.31%	5.27%	8.22%	8.61%

(a) ***, **, * indicate significance at the 1%, 5%, and 10% levels, respectively, and standard errors are in parentheses. (b) Estimated using Stata software

Among the five city heterogeneous control variables in the conditional *β* convergence estimation, economic development level (GDP) and government financial capacity (GFC) are significantly positive regardless of whether the spatial factor is included. This indicates that GDP and GFC is conducive to the improvement of the HRS in a specific city. The explanation is that faster economic development promotes residents' demand for public goods, including healthcare resources; meanwhile, it also enables specific cities to provide more fund to increase their supply [66]. These results are consistent with the findings of Cheng et al. [67]. However, the spatial effect coefficients of the above two factors are insignificant, indicating that the economic development level and government financial capacity do not have spillover effects on neighboring cities in HRS.

In addition, the urbanization level (UR) variable was only significant in the spatial panel model, indicating that the population agglomeration to urban areas within a specific city can help improve the allocation level of healthcare resources. However, the estimated coefficient was positive but insignificant in the model without spatial effects, reminding us to treat this result with caution. Finally, the estimated coefficients of city size (SIZE) and government financial self-sufficiency rate (GFS) are insignificant. However, some studies have reported that the impact of fiscal self-sufficiency rate is significantly positive with nationwide and provincial samples [30]. The reason may be that the fiscal self-sufficiency rate (average 44%) varies greatly among provinces in China, whereas the city-level fiscal self-sufficiency rate (average 68%) in the YRD is generally high, which weakens its impact on healthcare resource allocation. However, its spatial coefficient (W × ln_GFS) is significantly negative, suggesting that more empirical evidence is required.

In order to further explore the impact of the above-mentioned city heterogeneity variables on the *β* convergence rate, we added their interactive terms with the HRS to the empirical models. For the sake of model identifiability, the panel model without spatial effects is used for estimation. The results in Table 6 once again confirm the positive effects of economic development level (GDP) and government financial capacity (GFC) on HRS. GDP does not affect the convergence rate; whereas GFC significantly increases the convergence rate. In addition, the impact of government financial self-sufficiency (GFS) and city size (SIZE) is still insignificant, but this result should be interpreted in light of the fact that city size significantly inhibits the convergence rate in the YRD considering the significantly positive results of ln_SIZE × β. It highlights the negative impact of city size expansion on the balanced allocation of healthcare resources, which needs further empirical evidence.

**Table 6:** The impact of city heterogeneity variables on *β* convergence

Model	(5)	(6)	(7)	(8)	(9)	(10)
β	-1.258*** (0.121)	-0.659*** (0.153)	-0.713*** (0.184)	-0.332*** (0.085)	-0.775*** (0.094)	-0.720** (0.296)
ln_SIZE	0.086 (0.066)	-0.067 (0.062)	-0.064 (0.062)	-0.069 (0.060)	-0.068 (0.061)	0.052 (0.071)
ln_GDP	0.284*** (0.063)	0.219*** (0.069)	0.215*** (0.0640)	0.215*** (0.063)	0.222*** (0.064)	0.231*** (0.085)
ln_UR	0.228*** (0.077)	0.192** (0.079)	0.232** (0.115)	0.215*** (0.078)	0.173** (0.080)	0.290 (0.203)
ln_GFC	0.168*** (0.046)	0.137*** (0.049)	0.140*** (0.049)	0.024 (0.055)	0.161*** (0.050)	0.047 (0.076)
ln_GFS	-0.042 (0.049)	-0.006 (0.055)	-0.001 (0.054)	0.050 (0.052)	0.076 (0.072)	-0.036 (0.093)
ln_SIZE × β	0.094*** (0.017)					0.079*** (0.019)
ln_GDP × β		0.003 (0.016)				-0.025 (0.039)
ln_UR × β			0.024 (0.050)			0.024 (0.104)
ln_GFC × β				-0.117*** (0.031)		-0.098** (0.046)
ln_GFS × β					0.042* (0.025)	-0.012 (0.061)
N	492	492	492	492	492	492
R^2^	0.607	0.580	0.580	0.593	0.583	0.613

(a) ***, **, * indicate significance at the 1%, 5%, and 10% levels, respectively, and standard errors are in parentheses. (b) Estimated using Stata software

## Robustness test

Considering that different weight matrices may impact spatial panel model estimation [68], and the presence or absence of medical school in a city also matters for healthcare resource supply, we need to test the robustness of the above empirical results. We perform two different tests: (1) transforming the spatial weight matrix, and (2) distinguishing whether there are medical schools or graduate programs. Because the spatial inverse distance weight matrix used in the previous paper may be affected by the geographic area of a particular city, the distance between two adjacent cities may be greater than the distance between non-adjacent cities; therefore, we first use the spatial contiguity weight matrix to test the robustness (Table 7). It can be seen that the convergence coefficients are still significantly negative and the spatial effect coefficients are significantly positive. Meanwhile, the estimated coefficients are similar to those reported in Table 5. These results show that the estimates obtained by using spatial inverse distance matrix are robust.

**Table 7:** Robustness test results of transformation space weight matrix

Model	Spatial absolute β convergence	Spatial conditional β convergence
	two-way fixed effects SEM	two-way fixed effects SDM
β	-0.469*** (0.027)	-0.637*** (0.033)
W × β		0.152** (0.076)
ρ/λ	0.371*** (0.057)	0.347*** (0.058)
N	492	492
R^2^	0.284	0.195
Convergence rate	5.27%	8.44%

(a) ***, **, * indicate significance at the 1%, 5%, and 10% levels, respectively, and standard errors are in parentheses. (b) Estimated using Stata software

Now we differentiate subsamples for cities to perform robustness test. Table 8 shows that under different subsamples, the estimation coefficients of absolute *β* convergence and conditional *β* convergence are both significantly negative, indicating the robust convergence in the YRD region. Moreover, significant spatial effects are found in different models again. Furthermore, the spatial effect of medical-school subsamples is significantly negative, indicating that cities with medical schools have a resource agglomeration effect, which weakens HRS in neighboring cities. When narrowing the sample size to cities with medical graduate education, similar results are obtained. Since the agglomeration may be related to urban population size, according to the State Council [69], Type I (≤ 5 million) and Type II (> 5 million) subsamples are used for further estimation (Table 14 in Appendix). Robust results are obtained again for the convergence and spatial effects.

**Table 8:** Robustness test results of different medical education subsamples

Model	Absolute β convergence			Conditional β convergence		
	(11)	(12)	(13)	(14)	(15)	(16)
	No medical school	Medical school	Graduate education	No medical school	Medical school	Graduate education
β	-0.446*** (0.032)	-0.568*** (0.053)	-0.618*** (0.058)	-0.609*** (0.041)	-0.700*** (0.0552)	-0.797*** (0.069)
W × β				-0.080 (0.241)	-0.810* (0.456)	-1.321*** (0.491)
ρ/λ	0.345** (0.156)	-0.592** (0.262)	-1.070*** (0.281)	0.322** (0.158)	-0.847*** (0.284)	-1.143*** (0.293)
N	276	216	168	276	216	168
R^2^	0.400	0.105	0.111	0.082	0.141	0.100
Convergence rate	4.92%	6.99%	8.02%	7.83%	10.03%	13.29%

(a) ***, **, * indicate significance at the 1%, 5%, and 10% levels, respectively, and standard errors are in parentheses. (b) Estimated using Stata software

## Impact of healthcare reform policies

We now investigate the impact of the latest healthcare reforms. Since the impact may have a jump effect at the start of the reform, and it may also affect the convergence rate after the initiation of the reform, the dummy variable and its interaction are introduced to the empirical model. Table 9 indicates that the descending healthcare resources reform does not produce a robust jump effect with a negative but sometimes insignificant sign. It also does not significantly affect the convergence rate of HRS. This result is robust for both absolute and conditional *β* convergence estimation. However, this result should not be interpreted as the descending healthcare resources reform being ineffective. On the contrary, some studies have found that this reform is effective in promoting patients to choose low-level hospitals [65].

**Table 9:** Estimation results of *β* convergence with the descending healthcare resources reform

Model	Absolute β convergence				Conditional β convergence			
	(17)	(18)	(19)	(20)	(21)	(22)	(23)	(24)
β	-0.478*** (0.048)	-0.476*** (0.026)	-0.478*** (0.048)	-0.475*** (0.026)	-0.626*** (0.054)	-0.644*** (0.033)	-0.628*** (0.054)	-0.643*** (0.034)
DHR	-0.044** (0.019)	-0.044** (0.018)	-0.063 (0.072)	-0.076 (0.052)	-0.028* (0.016)	-0.031* (0.018)	-0.001 (0.061)	-0.076 (0.056)
DHR × β			-0.019 (0.057)	-0.032 (0.048)			0.026 (0.051)	-0.044 (0.051)
W × β						-0.058 (0.277)		-0.076 (0.278)
ρ/λ		0.346** (0.155)		0.358** (0.154)		0.309* (0.162)		0.312* (0.162)
N	492	492	492	492	492	492	492	492
R^2^	0.533	0.278	0.533	0.277	0.582	0.000	0.582	0.008

(a) ***, **, * indicate significance at the 1%, 5%, and 10% levels, respectively, and standard errors are in parentheses. (b) Estimated using Stata software

We also need to estimate the impact of the comprehensive medical reform launched in 2015 (Table 10). It can be seen that although there is some evidence that the reform dummy has a negative impact on the improvement of HRS, this effect becomes insignificant when the interaction term with *β* is added. The interaction term is insignificant in both absolute *β* convergence and conditional *β* convergence estimation. These results do not offer robust evidence for the impact of this reform on healthcare resource allocation, which is consistent with the conclusion of Wang [70].

**Table 10:** Estimation results of *β* convergence with the comprehensive medical reform

Model	Absolute β convergence				Conditional β convergence			
	(25)	(26)	(27)	(28)	(29)	(30)	(31)	(32)
β	-0.470*** (0.047)	-0.469*** (0.026)	-0.470*** (0.046)	-0.468*** (0.026)	-0.625*** (0.053)	-0.638*** (0.033)	-0.628*** (0.054)	-0.638*** (0.034)
CMR	-0.062*** (0.019)	-0.063*** (0.020)	-0.073 (0.065)	-0.083 (0.054)	-0.056*** (0.018)	-0.054** (0.024)	-0.019 (0.056)	-0.060 (0.055)
CMR × β			-0.011 (0.058)	-0.021 (0.053)			0.039 (0.051)	-0.007 (0.055)
W × β						-0.136 (0.272)		-0.145 (0.275)
ρ/λ		0.330** (0.158)		0.335** (0.158)		0.296* (0.164)		0.297* (0.164)
N	492	492	492	492	492	492	492	492
R^2^	0.536	0.281	0.536	0.280	0.587	0.028	0.588	0.035

(a) ***, **, * indicate significance at the 1%, 5%, and 10% levels, respectively, and standard errors are in parentheses. (b) Estimated using Stata software

## Discussion

The imbalanced allocation of healthcare resources is a common challenge around the world. However, such imbalance in China mainly occurs between core (usually provincial capital) cities with high-level hospitals and peripheral cities with vast rural areas and low-level hospitals, which is characterized by the coexistence of congestion of high-level hospitals and idle resources of low-level ones [5]. Over the past two decades, the Chinese government began to increase investment in low-level hospitals and promote the inflow of medical human capital to low-level hospitals, so as to promote the equalization of healthcare resource allocation. However, previous studies mainly used provincial-level data and ignored the spatial correlation [29, 56, 57]. For the first time, we use the data of prefecture-level cities in representative YRD region to evaluate the spatial HRS distribution and convergence in China, providing empirical evidence on China’s evolving healthcare resources allocation during the past decades.

Our empirical results show that the HRS in 41 cities at and above the prefectural in the YRD increases significantly, and the results of Gini coefficient and variation coefficient (*σ* convergence) show that the imbalance has significantly decreased, which is similar to the results of Chen and Han [22]. Although different cities with one province witnessed the reduced difference of HRS, the provincial differences are still keeping obvious. Since the HRS in China is mainly financed from local government below the provincial level, it is difficult to narrow the differences between provinces only through efforts of local governments, and this is still an important problem to be solved in China's future healthcare reform.

The convergence estimation of the HRS further supports the above-mentioned conclusion that the healthcare resource allocation in the YRD region has been improved. The absolute *β* convergence estimation indicates a more stable equilibrium trend within the YRD, however, the conditional *β* convergence estimation shows that intercity heterogeneity has a significant effect on the convergence rate. This conclusions are still robust when changing the spatial weight matrix and differentiating the regression subsamples. We also found that cities with stronger government financial capacity and medical education have a higher rate of convergence. The reason is that, the improvement of government financial capacity means that local governments have more financial funds to invest in public goods such as healthcare resources, which is helpful to promote convergence between cities [63]. Besides, cities with medical education have long-term accumulated advantages of medical education resources agglomeration, which can promote the HRS of the city by providing human capital supply for the medical market of a specific city.

The results of Moran’s *I* and LISA cluster maps report the significant spatial positive correlation of HRS in the YRD region, and the agglomeration and diffusion effect of different cities in geographical space, which suggests that neglecting the spatial effect will produce biased estimation results. Furthermore, our spatial panel model estimation also found significant spatial spillover effects of healthcare resource allocation in the YRD region, which was supported by a nationwide study by Xin [30]. However, it should be noted that if a specific city has medical schools or provides medical graduate programs, it will have a negative spatial spillover effect on its neighboring cities, again indicating the resources agglomeration effect of medical education.

In recent years, China introduced the descending healthcare resources reform and comprehensive medical reform. The existing literatures believed that these reforms are conducive to more balanced healthcare resources allocation by using provincial-level data or micro-data of community-level hospital [24, 71], other literatures believed that these reforms have little impact on it [21]. In this paper, we take the reform dummy into the model to estimate its convergence effect. We found that the recent reforms do not exert a significant impact on the HRS convergence. The explanation is that, the descending healthcare resources reform mainly encouraged the flow and spillover of human capital from (urban) high-level hospitals to low-level ones through government orders and cost subsidies, and its policy effect focused on the demand side by attracting patients to low-level hospitals. Thus, the impact of the descending healthcare resources reform is very different from the reform launched in 2003, which aimed to improve the infrastructure of low-level hospitals. While the comprehensive medical reform focuses on reforms like medical service prices, healthcare worker’s salary, and medical insurance, so its impact on healthcare resources supply is relatively weak.

From the perspective of international research, this study can provide some useful implications. First, we recognize that the healthcare resources allocation requires financial growth in terms of healthcare expenditures as well as increased human, and material resources, measured by indicators like numbers of doctors and beds. Compared with previous studies, this paper used the entropy weight method to construct a healthcare resource supply index from three dimensions of human, material, and financial resources, which can measure the healthcare resources allocation in a specific region more comprehensively. Secondly, our empirical results demonstrate the existence of significant spatial effects among neighboring regions, suggesting that spatial correlation should be fully considered in the study of inter-regional healthcare markets within a country. Finally, we added healthcare policy variables to the traditional convergence regression model, which could provide an alternative methodology in estimating the policy effects of healthcare reform and thus improve our understanding of inter-regional convergence driven by healthcare reforms.

It is necessary for us to report the limitations of this study. First, although we construct the HRS index from multiple dimensions based on available data, no human capital factor is included in the index. Therefore, the supply index may not fully reflect the qualitative change of resources supply. Second, the close spatial connection and convergence in the YRD could reflect the effect of China's healthcare resources reform during the past decades. However, if data are available in the future, this study should be extended to all provinces and different city clusters in China, to reflect the huge differences among cities throughout the country. Finally, both the descending healthcare resources reform and comprehensive medical reform mainly focus on the demand side, and therefore, they do not have a significant impact on the convergence of HRS. Future studies should collect demand-side data to construct a demand index and evaluate the spatial distribution and convergence of demand in China. Such efforts will deepen our understanding of the effect of China's latest healthcare reforms.

## Conclusions and policy implications

This paper focuses on one representative region, the YRD, and incorporates the spatial correlation factors to explore the spatial pattern and convergence of China's healthcare resource allocation in the context of the latest healthcare reforms. We use multi-index measurement and spatial panel regression technology to estimate the spatiotemporal evolution and spatial correlation dynamics of healthcare resource allocation in the YRD. This study also includes city heterogeneity factors and healthcare reforms and estimates their marginal effects.

It is found that during the sample period from 2007 to 2019, HRS in the YRD increased significantly and converged rapidly. Meanwhile, significant spatial correlation and agglomeration existed between provinces and cities, and the estimation results all indicated the accumulation of advantages of specific cities and their resource agglomeration effect in regional integration. In addition, different from the Chinese government's stated reform goal of promoting balanced allocation of resources, our empirical study did not find robust evidence that the latest reforms, including the descending healthcare resources reform and comprehensive medical reform, could help improve the convergence rate of healthcare resources in the YRD.

This study demonstrates that in the context of sustained and rapid economic growth, increased long-term investment in infrastructure and human resources in grassroots healthcare institutions, initiated in 2003, had the effect of promoting HRS and convergence between core and peripheral cities. In this convergence process, identifying regions with low resource supply levels and slower convergence rates, and then providing targeted support are important for future reforms. City heterogeneity, characterized by population size and differences in medical education, has a differentiated influence on the convergence rate and produces different spatial effects, which also highlights that city authorities should pay attention to the prominent role of high-level hospitals and medical education in the agglomeration of healthcare resources.

## Data Availability

The dataset used in this paper is available upon request.

## Appendix

Figure 4, Tables 11, 12, 13 and 14.

**Table 11:** Measurement results of the MRS from 2007 to 2019

City	2007	2009	2011	2013	2015	2017	2019
Shanghai	0.347	0.401	0.407	0.500	0.571	0.630	0.765
Hangzhou	0.248	0.285	0.359	0.416	0.483	0.590	0.630
Ningbo	0.225	0.252	0.348	0.391	0.445	0.465	0.543
Wenzhou	0.145	0.161	0.291	0.323	0.404	0.440	0.494
Jiaxing	0.195	0.229	0.271	0.289	0.358	0.398	0.436
Huzhou	0.180	0.211	0.252	0.299	0.374	0.419	0.514
Shaoxing	0.162	0.195	0.284	0.324	0.386	0.438	0.513
Jinhua	0.152	0.182	0.304	0.358	0.421	0.453	0.514
Quzhou	0.133	0.175	0.280	0.325	0.415	0.490	0.533
Zhoushan	0.256	0.318	0.375	0.413	0.525	0.543	0.573
Taizhou	0.133	0.166	0.247	0.284	0.360	0.401	0.464
Lishui	0.142	0.201	0.301	0.351	0.455	0.522	0.606
Nanjing	0.251	0.265	0.317	0.370	0.443	0.490	0.646
Wuxi	0.241	0.219	0.314	0.370	0.425	0.497	0.601
Xuzhou	0.109	0.142	0.193	0.346	0.393	0.461	0.506
Changzhou	0.197	0.224	0.243	0.313	0.387	0.424	0.477
Suzhou	0.175	0.196	0.271	0.330	0.383	0.454	0.534
Nantong	0.234	0.200	0.294	0.342	0.387	0.461	0.502
Lianyungang	0.090	0.121	0.232	0.282	0.334	0.398	0.448
Huai'an	0.083	0.120	0.251	0.307	0.349	0.420	0.435
Yancheng	0.087	0.107^*^	0.201	0.279	0.349	0.405	0.413
Yangzhou	0.187	0.171	0.247	0.291	0.337	0.405	0.422
Zhenjiang	0.169	0.183	0.235	0.291	0.622	0.357	0.391
Taizhou	0.124	0.141	0.260	0.370	0.389	0.426	0.481
Suqian	0.070	0.103	0.234	0.262	0.351	0.419	0.461
Hefei	0.136	0.215	0.255	0.291	0.340	0.419	0.486
Wuhu	0.168	0.219	0.251	0.293	0.364	0.423	0.430
Bengbu	0.105	0.181	0.279	0.322	0.361	0.389	0.442
Huainan	0.175	0.223	0.349	0.391	0.406	0.381	0.415
Ma'ansha	0.167	0.230	0.246	0.284	0.337	0.368	0.396
Huaibei	0.182	0.231	0.311	0.312	0.355	0.372	0.406
Tongling	0.211	0.256	0.336	0.389	0.440	0.369	0.415
Anqing	0.096	0.137	0.233	0.275	0.362	0.349	0.401
Huangshan	0.212	0.201	0.273	0.310	0.365	0.412	0.442
Fuyang	0.049	0.125	0.262	0.288	0.339	0.399	0.442
Suzhou	0.060	0.137	0.222	0.265	0.318	0.354	0.359
Chuzhou	0.073	0.137	0.201	0.274	0.320	0.361	0.429
Lu'an	0.066	0.141	0.217	0.254	0.363	0.353	0.381
Xuancheng	0.100	0.159	0.227	0.315	0.340	0.386	0.429
Chizhou	0.116	0.162	0.227	0.309	0.343	0.352	0.397
Haozhou	0.048	0.116	0.228	0.250	0.304	0.336	0.400

* means two-year moving average interpolation of the number of registered nurses because of the missing of data in 2009

**Table 12:** The healthcare reforms and medical education in the Yangtze River Delta, China

City	Descending healthcare resources reform	Comprehensive medica reform	Representative undergraduate medical school	Graduate education
Shanghai	2011.01	2016.02	Shanghai Jiao Tong University (earlier than 2007)	Yes
Hangzhou	2013.08	2017.03	Zhejiang University (earlier than 2007)	Yes
Ningbo	2013.08	2016.10	Ningbo University (earlier than 2007)	Yes
Wenzhou	2011.09	2016.12	Wenzhou Medical University (earlier than 2007)	Yes
Jiaxing	2011.10	2016.10	Jiaxing University (earlier than 2007)	No
Huzhou	2013.10	2016.10	Huzhou University (earlier than 2007)	No
Shaoxing	2010.12	2016.08	Shaoxing University (earlier than 2007)	No
Jinhua	2013.09	2016.09	No	No
Quzhou	2015.04	2017.02	No	No
Zhoushan	2014.01	2016.08	No	No
Taizhou	2013.09	2016.10	Taizhou University (earlier than 2007)	No
Lishui	2013.10	2016.12	Lishui University (2010)	No
Nanjing	2013.05	2015.10	Nanjing Medical University (earlier than 2007)	Yes
Wuxi	2014.12	2015.04	Wuxi School of Medicine, Jiangnan University (2012)	No
Xuzhou	2015.10	2015.10	Xuzhou Medical University (earlier than 2007)	Yes
Changzhou	2013.07	2015.08	Changzhou University (2011)	No
Suzhou	2015.03	2015.05	Soochow University (earlier than 2007)	Yes
Nantong	2015.07	2015.07	Nantong University (earlier than 2007)	Yes
Lianyungang	2016.01	2015.12	Nanjing Medical University (2013)	No
Huai'an	2015.09	2016.05	No	No
Yancheng	2015.10	2015.05	No	No
Yangzhou	2015.04	2015.08	Yangzhou University (earlier than 2007)	Yes
Zhenjiang	2015.05	2015.02	Jiangsu University (earlier than 2007)	Yes
Taizhou	2015.10	2016.03	Nanjing University Of Chinese Medicine (2010)	No
Suqian	2016.06	2017.12	No	No
Hefei	2014.12	2015.03	Anhui Medical University (earlier than 2007)	Yes
Wuhu	2016.09	2015.05	Wannan Medical College (earlier than 2007)	Yes
Bengbu	2014.08	2015.10	Bengbu Medical College (earlier than 2007)	Yes
Huainan	2014.08	2015.03	Anhui University of Science and Technology (earlier than 2007)	Yes
Ma'ansha	2015.04	2015.03	No	No
Huaibei	2014.10	2015.03	No	No
Tongling	2014.06	2015.06	No	No
Anqing	2015.04	2015.04	No	No
Huangshan	2015.12	2015.03	No	No
Fuyang	2015.04	2015.04	No	No
Suzhou	2015.04	2015.03	No	No
Chuzhou	2015.04	2015.04	No	No
Lu'an	2015.05	2015.03	No	No
Xuancheng	2016.06	2015.03	No	No
Chizhou	2015.06	2015.03	No	No
Haozhou	2015.05	2015.03	No	No

Source: The authors’ collection

**Table 13:** Results of LISA cluster in the Yangtze River Delta, China

Year	Agglomeration relation	The number of core city	List of core cities
2007	HH	4	Shanghai, Suzhou, Jiaxing, Zhoushan
	LL	5	Xuzhou, Lianyungang, Bengbu, Huai'an, Suqian
	LH	1	Xuancheng
	HL	2	Huaibei, Huainan
2013	HH	3	Nantong, Suzhou, Zhoushan
	LL	2	Bengbu, Huai'an
	LH	1	Jiaxing
	HL	3	Xuzhou, Nanjing, Huainan
2019	HH	4	Suzhou, Quzhou, Jinhua, Zhoushan
	LL	4	Fuyang, Bengbu, Anqing, Chizhou
	LH	1	Jiaxing
	HL	3	Hefei, Nanjing, Xuzhou

H/L represents the high/low supply of healthcare resources respectively, including four clustering relationships (HH, LL, LH and HL)

**Table 14:** *β* convergence regression results for different city size subsamples

Model	Absolute β convergence		Conditional β convergence	
	(1)	(2)	(3)	(4)
	Type I	Type II	Type I	Type II
β	-0.467*** (0.029)	-0.498*** (0.067)	-0.631*** (0.035)	-0.975*** (0.132)
ρ/λ	0.354** (0.154)	-0.872*** (0.296)	0.311* (0.161)	-0.888*** (0.304)
W × β			-0.186 (0.267)	-1.678** (0.755)
N	384	108	384	108
R^2^	0.329	0.098	0.012	0.131

Type I and Type II denote cities with urban population ≤ and > 5 million, respectively

## References

[1] Gwatkin DR (2017). Trends in health inequalities in developing countries. Lancet Glob Health.

[2] Jakovljevic M, Timofeyev Y, Ekkert N (2019). The impact of health expenditures on public health in BRICS nations. J Sport Health Sci.

[3] Jakovljevic M (2016). Comparison of historical medical spending patterns among the BRICS and G7. J Med Econ.

[4] Chi FL (2020). Reform of public health governance system based on the idea of putting people’s health as top priority. Admin Reform.

[5] Sun ZS, Wang SH, Barnes SR (2016). Understanding congestion in China's medical market: an incentive structure perspective. Health Policy Plann.

[6] The State Council (2015). Guiding Opinions of the General Office of the State Council on Promoting the Construction of a Hierarchical Diagnosis and Treatment System.

[7] The State Council (2017). Guiding Opinions of the General Office of the State Council on Promoting the Construction and Development of Medical Treatment Combination.

[8] Nixon J (2001). Discussion paper 183 convergence of health care spending and health outcomes in the European union 1960–1995. Nurs Manage.

[9] Panopoulou E, Pantelidis T (2011). Convergence in per capita health expenditures and health outcomes in the OECD countries. Appl Econ.

[10] Traoré O (2021). Convergence in public health expenditure across the Sub-Saharan African countries: does club convergence matter?. Health Econ Rev.

[11] Apergis N (2017). Convergence of health care expenditures across the US states. Soc Indic Res.

[12] Youkta K, Paramanik R (2020). Convergence analysis of health expenditure in Indian states: do political factors matter?. GeoJournal.

[13] Zhang G, Zhang L, Wu S (2016). The convergence of Chinese county government health expenditures: capitation and contribution. BMC Health Serv Res.

[14] Liang D, Zhang DL, Huang JY, Schweitzer S (2016). Does rapid and sustained economic growth lead to convergence in health resources. Inquiry.

[15] Hu HS, Wu SQ (2019). A research on structural optimization of basic public service based on the improvement of sense of Gain. Financ & Trade Econ.

[16] Hu R (2016). Analysis of public medical resource allocation strategy from the perspective of supply side. New West.

[17] Du C, Zhu HP (2016). The evolution logic of China's urban medical and health system. Soc Sci China.

[18] Wang J, Wang XY (2021). Research on China’s integration of health services: policy evolution and theoretical mechanism. J Public Man.

[19] Jin ST, Li B, Yang YC (2021). Does economic development narrow the supply gap of basic medical and health resources among regions? –metrological test based on 287 urban panel data. World Reg Stud.

[20] Zheng JC (2019). Study on equilibrium of health resource allocation in China. Chin Heal Resour.

[21] Chen JQ, Xu S, Gao J (2020). The mixed effect of China’s new health care reform on health insurance coverage and the efficiency of health service utilisation. Int J Env Res Pub He.

[22] Chen ZY, Han YG (2021). The dynamic evolution and spatial difference of basic medical and health services supply. J Zhongnan Univ Econ Law.

[23] Cheng DY, Fu D, Li S (2019). Study of spatial distribution characteristics of primary medical and health service facilities in Wurumuqi. Med Soc.

[24] Yang YY, Fu MY (2019). Evaluation of system effect of graded diagnosis and treatment. Sta Decis.

[25] Zhang XX, Chen SR (2019). Quantitative evaluation of the implementation effect of hierarchical medical reform policy in China: a case study of Xiamen city. Fujian Tribune.

[26] Central Committee of the Communist Party of China, State Council (2019). Outline of the Regional Integration Development Plan of the Yangtze River Delta.

[27] Liang WJ, Tang YM (2018). Study of health resource spatial non-equilibrium allocation in China. Health Econ Res.

[28] Ma ZF, Yin SG, Qiao WY (2018). Spatial equilibrium state and its time evolution of medical health resource supply level in China. Sci Geogr Sin.

[29] Zhou Y, Yu YS (2018). Analysis of the distribution status and convergence path of health resources in China: based on provincial panel data. Chin Heal Econ.

[30] Xin CC, Li J, Yang CF (2020). Research on regional difference and spatial convergence of medical and health service supply in China. Chin J Popul Sci.

[31] Sun CZ, Liu YY, Chen LX (2010). The Spatial-temporal diaparities of water footprints intensity based on Gini coefficient and Theil index in China. Acta Ecol Sin.

[32] Hao YB, Pei QY, Lu F (2017). Study on the configuration fairness and the efficiency of health resource in China at the end of the “Twelfth Five-year”. Chin Heal Res.

[33] Wang YY, Li YY, Qin SS (2019). Research on equity of allocation of primary-level medical and health service resources in China based on agglomeration degree. Chin J Health Stat.

[34] Li B, Zhang JX (2017). Study on distribution of health resource allocation in Henan province from 2006 to 2014. Chin Heal Res.

[35] Kakwani NC. Income Inequality and Poverty: methods of Estimation and Policy Applications. Oxford: Oxford Univ Press; 1980.

[36] Dagum C (1997). A new approach to the decomposition of the Gini income inequality ratio. Empir Econ.

[37] Yang Y, Yao CQ, Zhang JS (2018). Spatial agglomeration analysis of the research talents in the city group in the middle reaches of Yangtze River. Geosp Inf.

[38] Zhang Y, Zhang C, Wang ZQ (2019). Research on the equity and influencing factors of health resource allocation in Xinjiang from 2004 to 2016: a comprehensive perspective based on "population fairness" and "geographical equity". Chin Health Serv Manage.

[39] Su BB, Liu SJ, Lu YJ (2021). Evaluation of human resource allocation of primary healthcare in China: based on agglomeration degree. Chin J Health Policy.

[40] Jiang S, Min R, Fang PQ (2017). The impact of healthcare reform on the efficiency of public county hospitals in China. BMC Health Serv Res.

[41] Zhang YQ, Wang GL (2019). Evaluating the service efficiency of China’s grass-roots medical and health institutions based on DEA and RSR methods. Chin Health Serv Manage.

[42] Yu JL, Yang SG (2021). Regional differences and influencing factors of China’s medical and health resource supply level. Stat Decis.

[43] Zhang T, Sun LQ, Li ST (2017). Analysis of equity and efficiency of public health resource allocation in China: based on HRAD and DEA. Chin J Health Policy.

[44] Zhang C, Zhao XH, Ni J (2020). Fairness analysis of health resources allocation in Sichuan province based on agglomeration degree. Mod Prev Med.

[45] Hu HM, Chen DW, Gao QS (2016). Evaluation on health resources allocation in Zhejiang based on agglomeration degree. Chin Health Econ.

[46] He JJ, Fu WS, Li Z (2019). Evaluation on agglomeration degree of the allocation of health human resources in Henan province. Med Soc.

[47] Yi M, Peng JC, Zhang L (2020). Is the allocation of medical and health resources effective? characteristic facts from regional heterogeneity in China. Int J Equity Health.

[48] Guo YX, Song GQ, Zhou RR (2018). Research on the current situation and equity of health resource allocation in Anhui province. Chin Heal Res.

[49] Huang ZK, Feng ZM, Zhang CY (2016). Equity of health resources allocation in Hainan province: an evaluation with Theil index. Chin J Publ Health.

[50] Su BL, Shi T (2014). Measurement of equalization of public health resource allocation based on spatio-temporal scale. Chin J Health Stat.

[51] Li GZ, Li CX (2018). Analysis on regional inequality and dynamic evolution of health expenditure in China. Stat Decis.

[52] Xie YY, Nian X, Ma HH (2018). Spatial distribution characteristics and influencing factors of health services in China. J Huazhong Norm Univ.

[53] Zheng WS, Jiang HX, Ai HR (2015). Analysis of regional inequalities of basic medical resources supply in China. Geogr Res.

[54] Huo QL, Tang XM, Wang HY (2021). Spatial distribution and accessibility of medical facilities in Liupanshan area. Sci Surv Mapp.

[55] Liu B, Ding PC, Qin JJ (2020). Research on the spatial pattern of medical services in Beijing based on POI data. J Baoji Univ Arts Sci.

[56] Pan J, Wang P, Qin XZ (2017). Disparity and convergence: Chinese provincial government health expenditures. PLoS ONE.

[57] Yu JN (2018). Growth and convergence analysis of total factor productivity of health resource in China. Stat Decis.

[58] Islam N (2003). What have we learnt from the convergence debate?. J Econ Surv.

[59] Elhorst JP (2015). Matlab software for spatial panels. Int Regional Sci Rev.

[60] Han F, Yang LG (2020). How does the agglomeration of producer services promote the upgrading of manufacturing structure. Manage World.

[61] Mao YN, Wang XW, Feng RH (2015). Study on screening hospital efficiency evaluation index based on DEA. Health Econ Res.

[62] Wu YY (2021). The Effect of fiscal decentralization on the local public health services supply. Health Econ Res.

[63] Yan YN, Tan JL (2016). Empirical measurement and influencing factors of public health equality: a provincial empirical data analysis from 2004 to 2013. Fujian Tribune.

[64] Ye J (2016). On the effect of urbanization on the equalization of provincial basic health care: base on six central provinces data. J Zhongnan Univ Econ Law.

[65] Sun ZS, Wang SH, Zhao HJ (2020). Does descending resources reform improve patient satisfaction and reshape choice of care providers?. Inquiry.

[66] Li JG, Li ZY, Zhu YF (2013). Equalization of basic public health services among counties: restrictive factors and public policies. Publ Fin Res.

[67] Cheng LH, Yang DG (2018). Spatio-temporal evolution characteristics of health resources mismatch in Urumqi-Changji region. J Univ Chin Acad Sci.

[68] Su CW, Deng ZB, Li LP (2021). Spatial pattern evolution and convergence of water eco-civilization development index in China. J Univ Chin Acad Sci.

[69] The State Council. (2014). Notice of the State Council on Adjusting the Standards for City Size Classification.

[70] Wang XY (2019). Evaluation of the effect of comprehensive medical reform policy. Mod Econ Res.

[71] Duan H, Zhang YN, Hou YC (2020). Research on the influence of the policy of medical alliance on the service ability of primary community medical and health institutions. J Gansu Admin Inst.

